# A Review on Additive Manufacturing of Micromixing Devices

**DOI:** 10.3390/mi13010073

**Published:** 2021-12-31

**Authors:** Marina Garcia-Cardosa, Francisco-Javier Granados-Ortiz, Joaquín Ortega-Casanova

**Affiliations:** Fluid Mechanics Group, School of Industrial Engineering, University of Málaga, 29071 Malaga, Spain; ma17gc@gmail.com (M.G.-C.); jortega@uma.es (J.O.-C.)

**Keywords:** additive manufacturing, mechanical micromixer, microfluidic devices, 3D printing, fluid mechanics

## Abstract

In recent years, additive manufacturing has gained importance in a wide range of research applications such as medicine, biotechnology, engineering, etc. It has become one of the most innovative and high-performance manufacturing technologies of the moment. This review aims to show and discuss the characteristics of different existing additive manufacturing technologies for the construction of micromixers, which are devices used to mix two or more fluids at microscale. The present manuscript discusses all the choices to be made throughout the printing life cycle of a micromixer in order to achieve a high-quality microdevice. Resolution, precision, materials, and price, amongst other relevant characteristics, are discussed and reviewed in detail for each printing technology. Key information, suggestions, and future prospects are provided for manufacturing of micromixing machines based on the results from this review.

## 1. Introduction

As stated on the ASTM ISO/ASTM52900:15 Standard Terminology for Additive Manufacturing (AM) [1], “Additive manufacturing is the general term for those technologies that based on a geometrical representation create physical objects by successive addition of material”. This technology offers great solutions in cases where traditional manufacturing reaches its technological limits [2]. One of the main advantages of use is that it provides the opportunity to produce an object with complex geometry in a short time period. Therefore, AM is often applied to Rapid Prototyping (RP), which is crucial for the assessment and testing of products [3]. It is important to point out that depending on the literature, there may exist some confusion about whether AM is RP or not. The authors of the present review suggest to read, for instance, [4] for further clarification. Thus, 3D printing is a group of AM technologies in which a 3D object is created by the superposition of successive layers of a specific material [5]. It is often said that this is the key of this technology, due to the fact that each layer is a thin cross-section of the object derived from the computer-aided design (CAD) data. The term RP is used in several fields to describe a process about the creation of a system or a piece before it has been released to commercialisation [6]. In other words, it is the process of creating an object in a faster way with the aim of obtaining a base model from which other models will be derived, including the final model or prototype. These terminologies are used by both engineers and consultants to refer to a development process with the goal of finding a solution, testing ideas, and getting feedback during the development process. Having said that, by generating a model using a three-dimensional CAD system, a 3D object can be manufactured without the need to have planned the process in advance [7]. In addition, this manufacturing technology has a great advantage over others, namely that it does not require complex and detailed analysis of the geometry that makes up the part. The user will have an advantage if they know the tools that can be used, that processes that should be followed, and additional methods to promote the correct finishing of the desired piece. However, AM technology simplifies the process of creating and producing 3D objects from CAD data [4]. Thus, researchers have been interested in applying AM technology in tissue engineering, anatomical modelling, prosthetics, medicine, pharmaceuticals [8,9,10,11], aerospace and automotive engineering [4,12], and the fabrication of fluidic devices for various applications [13,14,15,16].

This review aims to compile all the necessary information based on a deep literature review to understand the most well-known technologies used to print 3D fluidic micromixers with AM and what aspects must be taken into account throughout the AM process. Additionally, the considerations and decisions that the researcher needs to make will be explained with the aim of achieving optimal printing according to certain requirements. These will be in relation to measurements of the design process, how to achieve good precision and resolution, what materials to use, and available printers, amongst others. The manuscript is structured into different sections as follows. Section 2 introduces the wide range of AM techniques in 3D printing and outlines the most popular for printing at microscale. Section 3 describes aspects to take into account to print a fluid-based static micromixers. Section 4 specifies current and near-future tendencies in AM to play a key role in the manufacture of micromixers. Finally, the conclusions drawn from this review are given in Section 5.

## 2. Printing Technologies in AM

**Figure 1:**
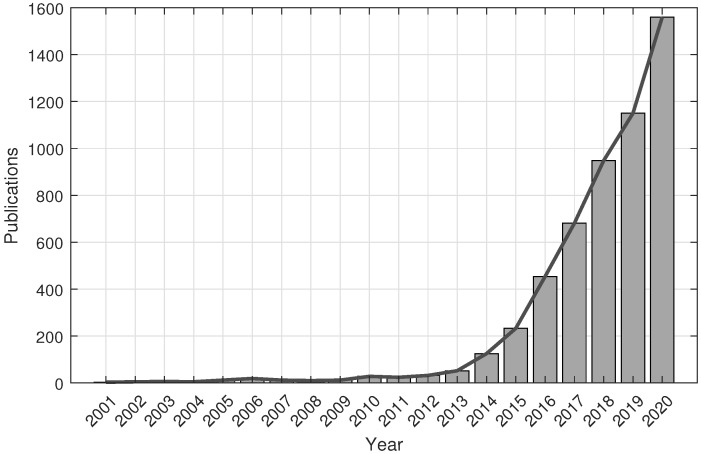
Publication trends. The bar graph shows the total number of publications per year from 2001 to 2020 (in Google Scholar: https://scholar.google.com/, accessed on 26 July 2021) with keywords: “3D printing”, “microfluidic” and “additive manufacturing”.

**Figure 2:**
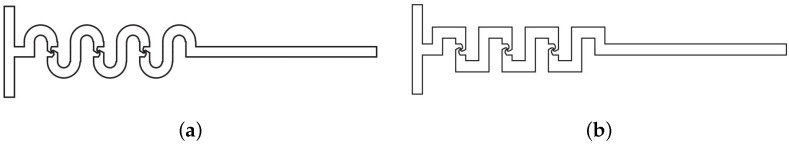
(**a**,**b**) Illustration of the structure complexity of different microdevices. (Reprinted from *Chemical Physics Letters*, Volume 734, Yao Chen and Xueye Chen, An improved design for passive micromixer based on topology optimization method, 136706, Copyright (2019), with permission from Elsevier).

**Figure 3:**
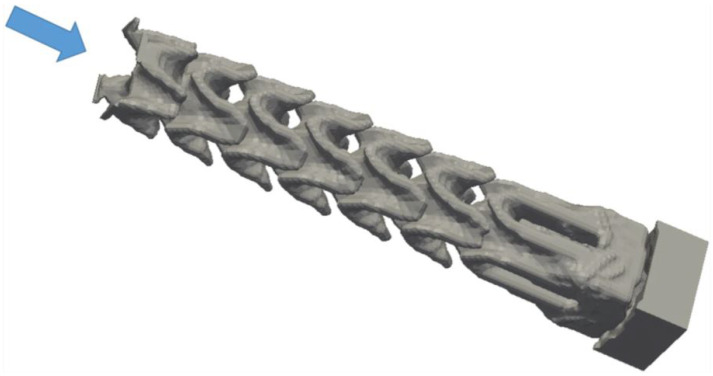
Illustration of the structure complexity of a microdevice in 3D. (Reprinted from *Structural and Multidisciplinary Optimization*, Volume 59, M. Pietropaoli, F. Montomoli and A. Gaymann, Three-dimensional fluid topology optimization for heat transfer, 801–812, Copyright (2019), with permission from Springer Open under the terms of the Creative Commons Attribution 4.0 International License: http://creativecommons.org/licenses/by/4.0/, accessed on 15 November 2021).

According to the American Society for Testing and Materials (ASTM) [17], the available AM technologies are classified into seven categories (Table 1), such that each category is developed under different processes [18,19]. All these processes share a common factor: the principle used of the modelling of layers. Moreover, depending on the type of technology used, one type of material or another may be used, such as polymers, metals, ceramics, and composites [5]. In recent years, AM has proven to be of great interest to society, as it can be seen in the graph in Figure 1. The figure illustrates how the number of investigations using 3D printing for microfluidic applications over the last few years has increased dramatically. However, in prototype microfabrication research, soft lithography is the most widely used and well-known technique [20], because it has enabled studies with low infrastructure costs and some ease of fabrication. Nevertheless, 3D printers capable of producing structures from a few microns to several centimetres are starting to stay one step ahead of soft lithography, because these printers have the ability to print in different materials with different properties. In addition, 3D printers offer the possibility to print a microfluidic micromixers in minutes and then make a model quickly and test its performance. For this reason, AM coupled with 3D printing has radically changed the way in which microdevices are manufactured. Figure 2 and Figure 3 show different designs of mixers for mass/heat exchange, and the complexity of their channels can be observed. Due to the complex shapes, AM is a relevant technology for their development, being often the only possibility.

Microfluidic devices offer a wide range of different applications, especially in both industrial and healthcare fields [21]. They comprise the science and technology of systems that handle and control small quantities of fluids. To perform their function, they make use of microscale structures [22]. There have been numerous investigations focused on using microfluidic devices fabricated by soft lithography, by using polydimethylsiloxane (PDMS) material, and even by micromoulding and injection moulding [23,24,25,26]. Soft lithography, however, is only capable of producing microdevices in 2.5D, so microscale parts to be used in 3D microfluidic applications are difficult to manufacture [27]. However, 3D printing offers many advantages over the aforementioned techniques. This is because the manufacturing of the model is done in a single step [28], so models can be manufactured with different materials which have different properties, depending on the potential application. In addition, this more rapid prototyping leads to a decrease in manufacturing time and also the fast integration capability of the manufactured products designs’ life cycles [29].

For many years, the goal has been to reduce the size and price of laboratory equipment by manufacturing small chips that were easy to use and easy to replicate. As a result, Lab-On-a-Chip (LOC) technologies have revolutionised many fields of knowledge, such as medicine, fluid mechanics, chemistry and biotechnology [30,31,32,33]. Nowadays, micromixers, as well as other fluidic microdevices, are used for different potential applications, which may or not be limited to particle concentration detection with an active dielectric mixer [34], glucose concentration detection with a passive circular mixer [35], or for dual quantitative detection of analytes with a passive Tesla mixer [36]. Applications more related to mechanical engineering may also include the mechanical characterisation and analysis of passive micro heat exchangers [37], heat transfer calculations and pressure drop measurements with a serpentine mixer [38], mixing efficiency and pressure drop for passive micromixer based on the topology optimization method [39], and plasma mixing analysis with a passive square-wave mixer [40]. These are microscale devices manufactured for the mixing of two fluid materials, usually achieved in microchannels with external turbulence and/or by the use of special microstructures [41]. Nevertheless, for the fabrication of these microfluidic devices, the well-known classical approaches are often time-consuming and complex. Traditional microfabrication involves the use of a cleanroom to fabricate a master with the help of a 2D photomask, followed by soft lithography and bonding [42]. Soft lithography was first introduced by Whitesides et al. [43,44] and has played a major role over the years in microfluidic fabrication, providing the ability to fabricate very precise and accurate microfluidic channels. As such, it is a very valuable tool. The process of carrying out microfabrication can be divided into two essential steps:

1. The generation of a master.
2. The use of the master to manufacture replicas.

For each lithography step, a mask writer is used to create chrome patterns on glass or silica plates [45].

Soft lithography consists of a family of techniques aimed at creating a soft polymeric mould, in most cases using PDMS from an original hard master mould, which is usually manufactured with photolithography in order to define a pattern. Moreover, the stamps are manufactured by curing PDMS on a master mould [46]. A silicon wafer, together with an epoxy-based photoresist (SU-8), is used to form the master of each layer. Thus, the moulded layers are created by melting the PDMS against the master mould [47]. In addition, PDMS is widely used to manufacture microfluidic channels due to its mechanical properties, since it is homogeneous, inexpensive, optically transparent, and non-toxic [47,48,49]. To cure this material, a mixture of silicone elastomer and a curing agent are usually poured onto the pattern and kept at a temperature of 70–80 °C for one hour. Other materials have also been used to fabricate the channels. With the use of soft lithography, micromixers that have structures lower than 10 nm in size can be manufactured [50]. Nevertheless, the need for experienced staff and the mechanical properties that the microdevices experience during their lifespan have limited the use of other materials [20].

It should be noted that a device will be considered readily manufacturable when it can be brought to market not only at low cost (meaning with cost such that including the incurred costs in terms of resources up to its final manufacture) but also when the manufacturing process is relatively fast and does not require investing in prohibitive technology or expensive handling [51]. However, it is difficult to achieve the above by making use of soft lithography, as there are obstacles that have made this technique less marketable:

- Many manual steps are needed to obtain the PDMS mould, making it difficult to fully automate the whole process.
- Chip inputs and outputs are prone to leakage and are not easy to connect [49,51].
- Control systems involve engineering skills and equipment that are not present in all laboratories.

This process includes various steps, in which labour-intensive processes are needed, making it difficult to rapidly innovate and these are crucial for new applications. These types of difficulties are found in each iteration of the manufacturing process, which require for instance printing a new photomask and lithography to produce a new master [52]. Due to this, in order to maximize manufacturing and minimize the total cost of the development of a micromixer, AM is leading new technological advances in this field [51].

Regarding the AM with 3D printing of micromixers, throughout the entire process (from design to the manufacturing of the printed part), some decisions have to be made. Several aspects must be analysed: the affordable price, the materials used to achieve certain properties, type of printer, model dimensions, required precision, etc. The choices made will determine the quality and costs of the final micromixing device.

**Figure 4:**
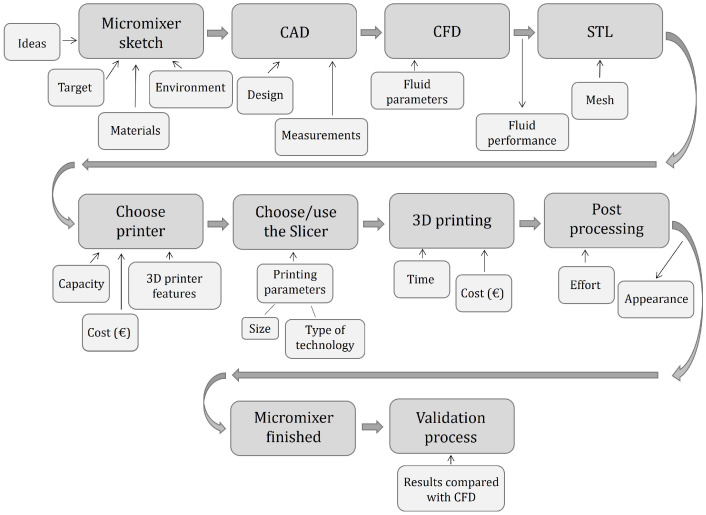
3D printing diagram assisted with CFD: a general design life cycle.

The 3D printing design life cycle is summarised in Figure 4. It shows the whole life cycle for printing a 3D object assisted with Computational Fluid Dynamics (CFD), as a generalization.

### 2.1. Relevant Characteristics of Each Category

Three-dimensional printing is defined as a set of manufacturing techniques based on the addition of material, aiming to manufacture complex parts and geometries more effectively and optimally than traditional methods. However, not all 3D printing techniques are suitable for the proper fabrication of microfluidic devices, such as micromixers. Nowadays, world-leading industrial countries are promoting 3D printing or AM as the technological basis for the manufacturing of the future, as the emergence of new materials and technological advances with AM has made it possible to manufacture 3D components previously unthinkable [4], developing printers that even allow the printing of several materials at the same time (multi-material) [53].

Despite new developments, as shown in Table 1, seven AM categories are currently commercially available on the market, each one with its own advantages and limitations, depending on the technology chosen and its corresponding purpose. In recent years, technological advances have helped AM to improve the ability to fabricate microfluidic devices [54,55]. Nowadays, microfluidic devices can be printed in a single step. In this way, AM replaces traditional cleanroom steps, making manufacturing more efficient and reducing costs. This facilitates rapid prototyping, making microfluidic technology more accessible for research in a variety of applications. In addition, innovation is sped up [56].

**Figure 5:**
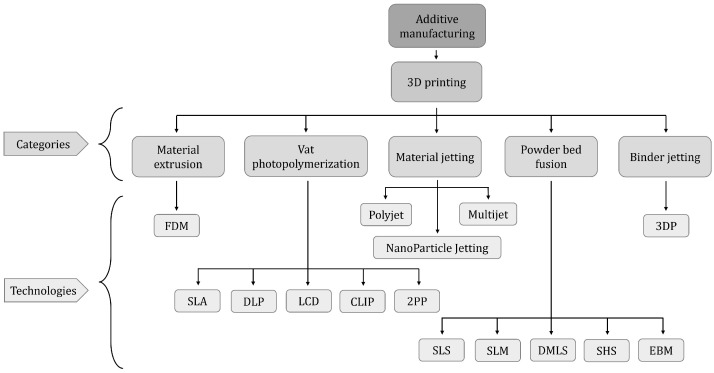
Five 3D printing categories with their respective technologies.

Within the above-mentioned categories, the most widely used technologies in the microfluidic field to print micromixers, so far, are the ones discussed in this review. These technologies belong to five different categories: material extrusion, vat photopolymerization, material jetting, powder bed fusion and binder jetting, as shown in Figure 5.

#### 2.1.1. Material Extrusion

In the late 1980s, Scott Crump invented the Fused Deposition Modelling (FDM) as a material extrusion technique [57]. This method was patented in 1989 [58], and it started to be commercialised in the early 1990s by Stratasys [59], which is a world leader in FDM technology and the leading manufacturer of industrial FDM systems. Stratasys was founded by Crump [60] and their wife, and due to the expiration of Crump’s patent, the diffusion of this technology boomed through the development of a wide variety of low-cost FDM machines [61,62].

For unfamiliar readers, FDM consists of extruding a molten filament of polymeric material to be deposited afterwards. The material used in the FDM process is a filament of polymeric material that is softened and melted with the help of heat and then extruded. The filament is pushed and fed through a nozzle with a specific small diameter and then deposited layer by layer on the printing bed [63]. The filament has a standard diameter, which can be either 1.75 mm or 2.85 mm, and is supplied on spools. The most common FDM printers have a standard Cartesian structure and an extrusion head. The filament is unwound from the spool with the help of a motor and a set of gears, knurled pulleys, and clamping screws that press and drive the filament in order to push it through a guide tube into the extrusion head. This process is performed as more material is needed to continue the construction of the 3D part. The extrusion head can have several extruders, each of them with a chamber that is heated, thus achieving the objective of softening the filament inside up to a certain viscosity, and then passing through the nozzle when pressure is applied [4]. The nozzle is interchangeable and also has standard-size diameters, which can vary from 0.3 mm to 0.6 mm, depending on the machine and/or the chosen manufacturing company [64,65]. It should be noted that the gap between the nozzle and the printer bed also corresponds to the thickness of the build layer, which is a factor to be taken into account when performing the calibration process before starting to print a part, as it is very important to establish the correct nozzle height and level the bed [66,67].

**Figure 6:**
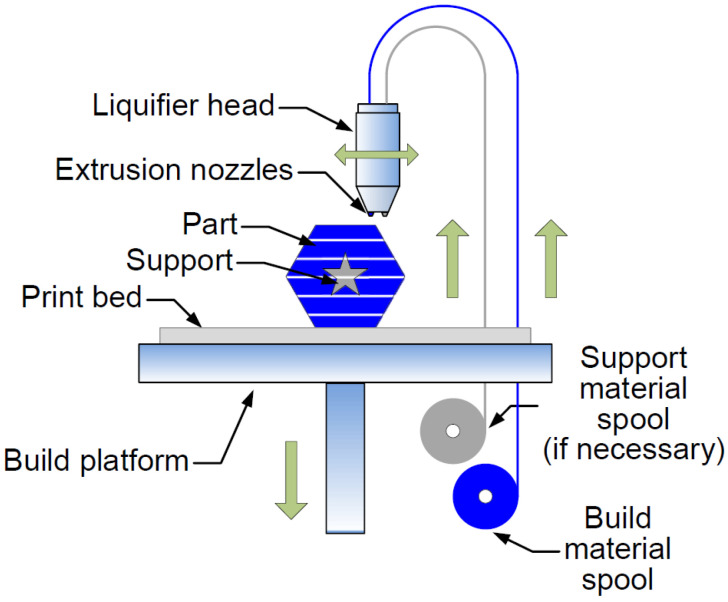
Example of FDM printer printing. (Reprinted from *Composites Part B: Engineering*, Volume 80, Fuda Ning, Weilong Cong, Jingjing Qiu, Junhua Wei, Shiren Wang, Additive manufacturing of carbon fiber reinforced thermoplastic composites using fused deposition modeling, 369–378, Copyright (2015), with permission from Elsevier).

Low-cost FDM machines are generally equipped with a single extruder and the print bed, which are the other necessary parts that comprise the machine at room temperature. In addition, their setup, such as calibration and material change, is usually manual. However, for industrial cases, the machines are equipped with a heated bed, together with advanced systems for improved positioning accuracy and faster extrusion head movement speed, which are often automatic, making material changeover faster. Industrial systems come with at least two extruders (see Figure 6), one of which is used to deposit the part building material, and the other to deposit the necessary support material (to support hollow shapes and protruding surfaces). When the part (printed object) is finished, the support material can be removed by immersing the construction for a few hours in a chemical bath that dissolves the built-up supports, leaving only the construction material as a final result [18].

Regarding the materials available for FDM technology [68], there are several ones such as acrylonitrile butadiene styrene (ABS), acrylonitrile styrene acrylate (ASA), polyamide (PA), polyethylene terephthalate with glycol (PET-G), polycarbonate (PC), polypropylene (PP), polyetherketoneketone (PEKK), polylactic acid (PLA), polyphenylsulfone (PPSF), and thermoplastic polyurethane (TPU), among others. Additionally, there are methods to improve the material properties of thermoplastic parts [69]. The most well-known materials are ABS and PLA [70]. Nonetheless, the popularity of PET-G, ASA, and PP is gradually increasing. The temperature recommended to work with these materials during the process [71,72,73,74,75] is indicated in Table 2, although they can vary depending on the specific composition of the material used [72]. As a general rule, it should be noted that the higher the extrusion temperature, the lower the viscosity. This leads to easier material flow and a higher deposition speed, although sometimes this is not always good, as it can damage the quality of the overlapping of the layers [72].

The surface roughness of FDM-fabricated parts can be profoundly affected by layer height, shape of the piece, and surface curvature with respect to the build orientation [76,77,78,79]. The ideal layer thickness will depend on the piece to be printed and its future application. For example, if the aim is to manufacture large parts and the final quality is not a priority, the choice will be to choose thick layers, and consequently the printing time will be shorter [80,81].

One of the great advantages of FDM technology is that it is one of the few AM technologies that allows the manufacture of parts with different materials in a single construction process through the use of more than one extruder [82,83]. This can be done with the use of an extruder containing soluble material to place supports that allow the manufacture of joint parts that have relative movement to form a system or assembly. This provides the correct tolerances and appropriate clearances to the coupled parts in the CAD model [84]. Moreover, the quality of the final result will depend on the orientation and layer thickness chosen for printing the CAD model, as well as its size [85,86].

The price of FDM machines can be less than EUR 1000, some being somewhat rudimentary but very affordable at EUR 90 or EUR 120, but, if the user desires to work with machinery for an industrial system with a working volume of half a cubic metre, which also offers a much higher quality, precision and accuracy, the price of FDM machines could exceed EUR 20,000 [87,88]. On the contrary, the commercial price of FDM materials is generally lower [89,90,91] than the price of materials used in other printing techniques. In addition, it is obvious that the expected price can vary depending on whether a specific composition or certain properties desired, for instance, if the required printing material is translucent.

In conclusion, FDM technology can be summarised as having the following advantages (+) and drawbacks (−):

+ FDM technology is the most cost-effective method when it comes to manufacturing customised thermoplastic parts and prototypes.
+ Manufacturing times are usually short.
+ There is a wide range of materials available, which are suitable for both industrial prototypes and some non-commercial functional applications.
− It has the lowest dimensional accuracy and resolution compared to other 3D printing technologies, so it is not suitable for parts with complex details. However, its cost is lower in comparison to other available technologies. It is used for products where details are not so important.
− Printed parts will have layer lines that will be visible, hence, if a smooth finish is desired, the piece will require a post-processing [92]. In addition, the bonding mechanism of the layers will make the parts inherently anisotropic. Therefore, this technology is not recommended for manufacturing parts that will be used as mechanically critical components.

Since 2009, this technology has had a great boom due to the expiration of a patent that boosted a significant increase in the use of this technology in industry, as for instance in the consumer market [93].

#### 2.1.2. Vat Photopolymerization

Following the development of photopolymers around 1960, Charles Hull began to investigate materials that were UV-cured by exposing them to a scanning laser, which was similar to the system currently used in laser printers. At the begining of the decade of the 1980, he found out that he could produce solid polymer patterns, and more importantly, he discovered that, by curing one layer on top of another, a solid 3D object could be produced within a few hours. This discovery led to the stereolithography (SLA/SL). He registered a patent, and when it was granted, he put the first stereolithography device on the market, together with the company called 3D Systems [94,95]. The term “stereolithography” was defined as a method for manufacturing solid objects by successively printing thin layers of a curable material using ultraviolet (UV) laser irradiation [96]. Regarding the stereolithography process, a platform located inside a tank containing the photopolymer moves the solid part downwards and the laser traces the next layer of uncured photopolymer. This process is called polymerization and is carried out until all the layers are finished forming the complete structure of the piece [97]. Stereolithography and other related processes are encompassed by the term “vat photopolymerization”.

The vat photopolymerization can be divided into two different process configurations that will determine how the printer works, plus an additional one that has attracted some research interest [94]:

- Vector/spot scanning: typical of SLA printers.
- Layer projection: irradiate entire layers.
- Two-photon approaches: high-resolution point-by-point approaches.

As mentioned above, vat photopolymerization is the term used to refer to a range of 3D printing technologies that work with laser or light that can come from different sources, to cure a polymer or polymerize a resin [98]. These technologies in the production of devices are characterised by their manufacturing process. The process they use is based on the conversion of liquid to solid, using computer spatially controlled photopolymerization to create solid objects from a vat of liquid resins under light irradiation [99]. The different resins used can have different light absorption spectra, and they have different penetration depths. Therefore, the same exposure parameters may lead to different results when using non-identical resins, and completely different structural behaviours may be observed [100]. The most common vat photopolymerization processes are stereolithography (SLA), digital light processing (DLP), liquid crystal display (LCD), continuous light interface production (CLIP), and two-photon polymerization (2PP).

The main differences amongst these vat photopolymerization technologies are as follows:

- SLA works with the light source of a laser beam. It is the most commonly used among the vat photopolmerization technologies. The laser can be located under or above the resin tank, and if the laser is located under the tank, this technology provides better results for small build volumes [101].
- DLP works with the light source of a digital light projector. DLP is similar to SLA, but the printing speed is faster due to the use of the projector, which cures each layer sequentially. This technology can make use of a Digital Micromirror Device (DMD) for the illumination [100], making it possible to rotate in a rapid way and reflect light.
- LCD works with Light-Emitting Diodes (LED). This technique is also called Daylight Polymer Printing (DPP), since it uses unmodified LCD screens and a daylight polymer. The main difference with DLP lies in the imaging system, because of the fact that this technology uses a liquid crystal display, which prevents light from passing through. Thus, the resolution of the liquid crystal display is very high; however, the accuracy of LCD is lower than that of DLP [102]. It is also faster than SLA technology.
- CLIP works with LEDs and oxygen. Thanks to the continuous liquid interface production method, this technology increases the printing speed and the resolution [103]. It works similarly to DLP. It has a “dead zone” to prevent the adhesion of the piece to the window, and this region is located between an oxygen permeable window and the curing part surface, which targets the inhibition of free radical photopolymerization [104,105].
- 2PP works with the light source of a titanium sapphire femtosecond laser. This method aims to achieve a resolution below the diffraction limit [106]. The fabrication process is defined by an objective lens focusing the laser onto the photosensitive resin [107], and inside the resin, the polymerization process takes place [108]. It is widely used in systems where there is a need to use nano and micro elements [109], because it has a subdiffraction-limit resolution down to 100 nm [110].

**Figure 7:**
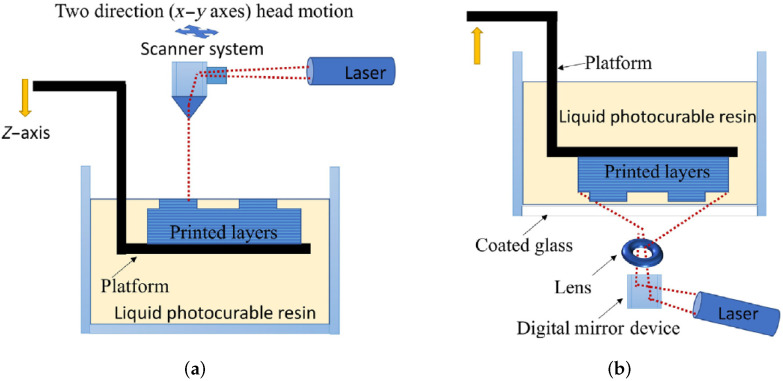
Illustration of the SLA and DLP technologies: (**a**) SLA and (**b**) DLP. (Reprinted from *Fiber-Reinforced Nanocomposites: Fundamentals and Applications*, 1st Edition, Dikshit, Vishwesh and Goh, Guo Dong and Nagalingam, Arun Prasanth and Goh, Guo Liang and Yeong, Wai Yee, Recent progress in 3D printing of fiber-reinforced composite and nanocomposites, 371–394, Copyright (2020), with permission from Elsevier).

**Figure 8:**
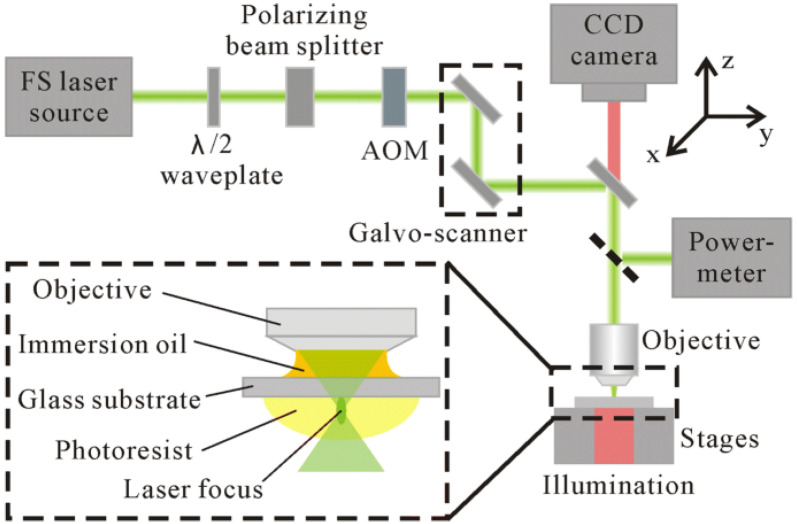
2PP technology. (Reprinted from *Nanoscale Research Letters*, Volume 14, Lei Zheng, Kestutis Kurselis, Ayman El-Tamer, Ulf Hinze, Carsten Reinhardt, Ludger Overmeyer and Boris Chichkov, Nanofabrication of High-Resolution Periodic Structures with a Gap Size Below 100 nm by Two-Photon Polymerization, 1–9, Copyright (2019), with permission from Springer Open under the terms of the Creative Commons Attribution 4.0 International License: http://creativecommons.org/licenses/by/4.0/, accessed on 15 November 2021).

As mentioned above, the LCD and CLIP technologies have emerged from DLP; hence, they have been developed as an improvement. Thus, it is usually admitted that the SLA and DLP are the most popular techniques. In Figure 7 and Figure 8 are shown the printing process of SLA, DLP, and 2PP technologies.

The curing light source and the light projection method play a key role in these technologies when assessing the accuracy of the printed models. In the case of SLA, the footprint of the curing laser beam is a spot, and the accuracy in this case will depend on its diameter. However, for DLP, LCD, and CLIP, the accuracy is provided by the pixel matrix, which is given by the resolution of the digital projector employed in these technologies [111]. Therefore, DLP, LCD, and CLIP technologies produce parts with lower accuracy than SLA. Thus, it can be seen that SLA technology can deliver higher accuracy but with longer production times. In addition, CLIP technology provides more accuracy than DLP [112], as was mentioned above, thanks to the continuous motion of its platform and the existence of the dead zone [113]. In micro-stereolithography technology, the conditions that are considered to be of greatest importance are the laser power, scanning speed, and exposure time [114]. They will influence the curing time and printing resolution [115].

If high precision and resolution are required, the most recommended technology is SLA printing, as it enables the fabrication of complex internal structures in detail. In addition, it has the ability to produce a larger number of objects in an estimated time frame. The advantages (+) and disadvantages (−) of said vat photopolymerization technologies are those specified below:

- SLA:
  + It is able to print large models with a high accuracy and surface finish.
  + The printing size is not limited.
  − High elapsed times.
- DLP:
  + Higher print speed in comparison to SLA.
  + Lower cost (low price) of printers.
  + Very good precision.
  − High cost of materials.
  − The projection size is limited.
- LCD:
  + Lower cost (low price) of printers.
  + Good resolution.
  − Short lifespan.
  − The intensity of the light is weak.
  − The liquid tank must be cleaned regularly (requires continuous maintenance).
- CLIP:
  + Higher print speed in comparison to DLP.
  − To achieve rapid printing, a low viscosity resin and a hollow model are necessary.
  − Efficiency is not high.
  − The use of the permeable oxygen membrane is expensive.
- 2PP:
  + It provides a high spatial resolution.
  + The laser is able to penetrate deeply into the material.
  − Expensive (price) printers.
  − Limited building area.
  − Errors in voxels affect the accuracy.
  − Low building speed.

After the curing process, the printed objects can be stable, hard, and even elastic, being able to withstand very low and high temperatures. This is because, depending on the type of chosen resin, the object will have different mechanical and chemical properties, which will make it suitable for specific applications. In general, photopolymer materials can be standard, structural, strong and durable, flexible, and elastic. Despite this, the standard resins that are commonly used have not been manufactured to meet certain specifications that are necessary in the engineering world, as others have, and the most common colours are black, grey, white, and transparent [116]. In addition, within the resins that encompass the standard resins, there is a draft resin, which can cure faster than the conventional one [117]. Durable resins are made of polyethylene (PE) or polypropylene (PP), which have high ductility and resistance to both deformation and impact. However, resins made with elastomeric polyurethane (EPU) provide elasticity and flexibility. Biocompatible resins are also available, which must undergo cytotoxicity testing and biological evaluation to ensure that they do not cause problems when they are in contact with the human body. Finally, if ceramic resin is desired, a photopolymer must be filled with silica [111].

One of the features that these technologies present is that many printing structures do not require any support material, since the unpolymerised material acts as support [118]. However, the printers can use perforated support structures, which are easily removed when the print is finished [119], and they are used to prevent deflection or movement of the piece caused by the gravity and the printing [120]. When the printing is finished, a post-processing must be carried out, and the first step is to remove the excess of resin by immersing the piece in a bath of isopropanol, and then the mechanical removal of the support structures is done. The last step of this post-processing will be to accomplish a final curing [121]. Nevertheless, if it has been used a draft resin, this final curing is not necessary. It should be noted that the materials used by these printers are not sealed, whereas the materials of Polyjet printers are. Hence, prior to curing, these materials are slightly toxic, owing to the fact that manufacturers recommend taking protective measures, such as wearing gloves and a mask when handling these resins [120].

In relation to the printer prices depending on the technology chosen, LCD 3D printers use components that are cheaper than other vat photopolymerization technologies, making them inexpensive resin 3D-printing solutions. This enables the user to have a wider range of manufacturers. Therefore, if the level of the resin 3D printer is for beginners, the LCD screen will be a good choice. However, if highest accuracy is desired in terms of details and good printing speeds, a professional DLP or SLA printer is recommended. In addition, if the objective is nanoscale printing, the best technology will be 2PP, but its price is the highest one amongst these technologies. The price range of vat photopolymerization printers is from EUR 115 [122] to more than EUR 250,000 [123,124].

#### 2.1.3. Material Jetting

Material Jetting (MJ) is a printing process included within AM, which is capable of printing on multiple materials during the same print job [125]. The method to create 3D objects is similar to a 2D jet printer (standard home/office printers) where the printing material is ink. The creation of 3D models is accomplished through the use of movable inkjet print heads. These heads inject a photopolymer onto a building platform. The photopolymer is injected by droplets that are selectively ejected as the heads move over the build area [126]. In inkjet printers, the material is injected using the Drop On Demand (DOD) or Continuous Ink Jet (CIJ) process. In the case of CIJ, lower viscosity fluids are used at a higher drop speed than in DOD, so it is often used when a high print speed is required. However, if higher precision is desired, it is better to use DOD [127].

MJ processes allow the printing of multi-material and degraded material structures. The printheads include several separate nozzles to be fed with different materials and to be controlled individually. The material, deposited initially as droplets in a liquid state, eventually solidifies. For this purpose, the most common method of carrying out this process is to cure the photopolymer ink with UV light, with each photopolymer layer being cured immediately after it has been injected, resulting in fully cured multi-material 3D parts [97].

There are several factors that can affect the quality of MJ, one of which is the shape of the deposited droplet, as this can affect resolution, precision, and even accuracy. Another factor is that, in order to get a correct print head sweep speed, the droplet splash and jetting frequency must be coordinated. Moreover, certain conditions must be taken into account for 3D printing with MJ [128,129,130]:

- Jetting parameters: frequency, magnitude, and width of the signal.
- Ink properties: surface tension and viscosity.
- Environment: pressure, humidity, and temperature.

**Figure 9:**
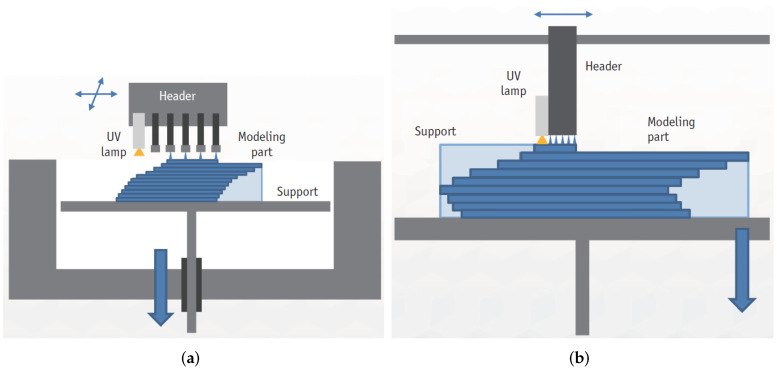
Examples of Polyjet and Multijet printers printing: (**a**) Polyjet and (**b**) Multijet. (Reprinted images from *Korean Journal of Radiology*, Volume 17, Kim, Guk Bae and Lee, Sangwook and Kim, Haekang and Yang, Dong Hyun and Kim, Young-Hak and Kyung, Yoon Soo and Kim, Choung-Soo and Choi, Se Hoon and Kim, Bum Joon and Ha, Hojin and others, Three-Dimensional Printing: Basic Principles and Applications in Medicine and Radiology, 182–197, Copyright (2016), with permission from The Korean Society of Radiology under the terms of the Creative Commons Attribution Non-Commercial License: http://creativecommons.org/licenses/by-nc/3.0, accessed on 15 November 2021).

Thanks to the use of this technology, it is possible to obtain 3D models or objects of different colours and hardnesses, with different properties and characteristics obtained within the same manufactured device [131,132,133]. During the manufacturing of these parts, it is necessary to use construction and support materials [132,134], although the latter may be removed. This action belongs to the post-processing and can be done manually (a few at the beginning, if it is possible) with the help of a water jet or by means of a chemical bath [135,136,137]. As with other AM technologies, the support material is not part of the final result, but it is necessary to use it because the building material must be properly deposited in voids or overhanging areas; see Figure 9. Furthermore, in MJ, the support material is a dense structure, and, most of the time, the amount of support material used is almost equal to the amount of building material, which makes this technology more expensive than other AM technologies [96]. MJ enables the manufacturing of parts with high resolution of 10–30 μm in layer thickness; hence, individual layers are not very visible [96,97,134] and are 42 μm in-plane [138].

The knowledge of the benefits provided by lithographic methods together with those obtained from MJ have led to the emergence of two major technologies that combine all these advantages: Polyjet and Multijet [97]. Both technologies are very similar; however, by analysing the post-processing, a remarkable difference can be found between Polyjet and Multijet. While Polyjet technology uses a photocurable material as support material, Multijet uses a wax. Due to this difference, the removal process of the support material differs between these two technologies [139]. Polyjet post-processing is shorter and simpler than that of Multijet [140].

The principal advantages (+) and disadvantages (−) of MJ technologies are:

+ Capability of printing multi-material in different colours and gradient structures.
+ Printers are able to build large structures with complex shapes and smooth finishes.
+ It provides high resolution: it is an attractive technology for microfluidic applications.
+ Polyjet printers are user-friendly.
− Printed devices need some post-processing.
− Ink deposition time is short, so several requirements in relation to viscosity and surface tension must be taken into account.
− Printers are expensive and proprietary.

Another type of MJ printing technology is NanoParticle Jetting (NPJ), a technology developed by XJet [141]. It uses metal or ceramic powder suspensions to build parts. Unlike Polyjet or Multijet, NPJ injects a liquid containing the nanoparticles of the material at the same time as the support material. With NPJ, high and superfine detail parts can be manufactured. This is accomplished in a heated bed; thus, particles adhere in all directions because of the evaporation of the liquid injected [141,142]. Additionally, the removal of the support material is effortless [143]. The greatest disadvantage of this technology is the high cost, which is more than EUR 250,000 [144]. Prices of Polyjet and Multijet technologies vary more: there are printers that are cheaper than those for NPJ. Nevertheless, they are expensive compared to aforementioned 3D categories in previous subsections, as prices range from EUR 19,000 [145] to more than EUR 250,000 [146].

#### 2.1.4. Powder Bed Fusion

**Figure 10:**
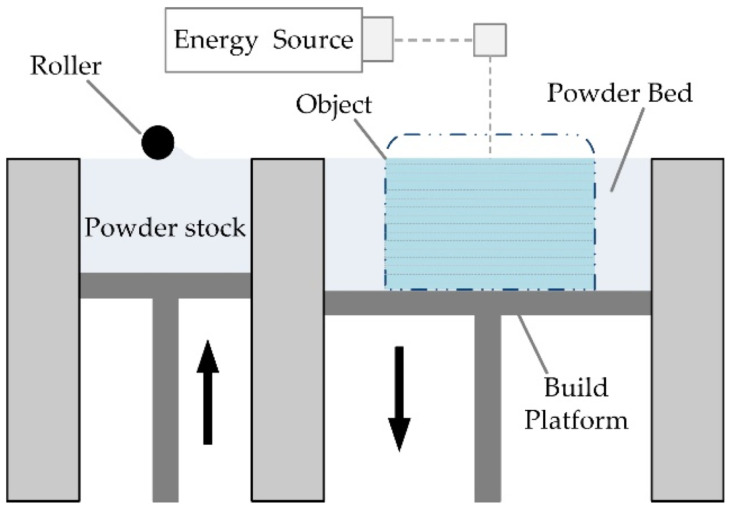
Illustration of the general PBF printing process. (Reprinted from *Metals*, Volume 9, Long Bai, Cheng Gong, Xiaohong Chen, Yuanxi Sun, Junfang Zhang, Lecai Cai, Shengyan Zhu and Sheng Quan Xie, Additive Manufacturing of Customized Metallic Orthopedic Implants: Materials, Structures, and Surface Modifications, 1004, Copyright (2019), with permission from *Metals*, MDPI, Basel, Switzerland, under the terms of the Creative Commons Attribution License: http://creativecommons.org/licenses/by/4.0/, accessed on 15 November 2021).

Powder Bed Fusion (PBF) encompasses a set of technologies that can be used for the manufacture of parts based on the concept of layer-by-layer addition [147]. In order to carry out the manufacturing process, lasers or electron beams are used as thermal energy sources for irradiation, fusion, and melting of powder particles [148]; see Figure 10. The materials popularly used in PBF printing processes are polymer [149] and metal powders [150].

In 1984, Carl Deckard, a student at the University of Texas, invented the first PBF technique, the Selective Laser Sintering (SLS) [151]. Deckard developed a machine that could create solid entities by fusing powder particles with the use of a powerful laser. This work was carried out under the supervision of Professor Beaman [152], and the technology was patented in 1990 [153]. Furthermore, the patent specified that the laser used would be less expensive than the CO_2_ laser [151]. Following this, the other PBF technologies were invented: selective laser melting (SLM), direct metal laser sintering (DMLS), selective heat sintering (SHS) and electron beam melting (EBM). The characteristics of each technology are specified as follows.

SLS is usually associated with polymer laser sintering. The powder is spread on the platform with the help of the roller mechanism when the piston moves upwards. The laser beam scans the powders selectively to synthesise the powder particles. In this way, the first layer is formed. The building area of the part moves downwards (according to the desired thickness), and the second layer is built on top of the already-synthesised first layer [154]. SLS technology provides an impression with a porous material recommended only for medical implants or non-wetted objects [155]. In the SLM, the manufacturing process is similar to the SLS one. When using metal powders or ceramics, SLM is usually applied [156]. It provides excellent mechanical properties and good precision [157]. The main difference between SLS and SLM is that SLS uses the laser to fuse small particles of raw material to build a 3D part, whereas SLM completely melts the powdered material with the laser, resulting in local melt pools, thus obtaining very dense parts (>99% density) [158]. The DMLS procedure is similar to the SLM working process. A laser is used, which is directly exposed onto the metal powder during liquid phase sintering. Parts are created by selectively melting thin layers with the use of the scanning laser beam [159]. SLM and DMLS differ in the powder bed temperature used to synthesise or melt the material [160]. Regarding SHS, this AM technology uses a heated head (touching the powder) to melt the plastic powder particles [161]. Finally, in EBM, the source of thermal energy for fusing metal powders is an electron beam [162]. Moreover, the electrons are emitted from a tungsten filament that is heated at high speed [163].

Support structures are essential for proper building of the objects, especially in certain areas that are critical during printing process, such as overhang areas. Support structures provide greater geometric integrity and aid the component to bond better to the substrate while layers are built up [164]. Nevertheless, the removal process of support structures can be tedious [165].

The principal advantages (+) and drawbacks (−) of PBF technologies are:

+ Solvent-free process: the unsintered powder acts as support.
+ SLS can produce 3D parts with high controlled porosity and high pore connectivity [166].
+ The unsintered powder material can be recycled and reused.
+ EBM provides better microstructural control.
+ They can be used to manufacture complex parts with little effort.
− A large quantity of powder is required to complete the printing process.
− A post-processing process may be required.
− The quality of metal parts printed with SLS is lower than that of parts printed with EBM.
− EBM technology is expensive because it requires vacuum operation.
− High power usage is required.

The price of PBF printers is very high. A SLS printer containing a small printing area (printing with thermoplastic polymer powder) can cost around 6000 € [167]. If a large printing area is necessary, then the price can go up to 444,000 € [168]. However, PBF printers can be even more expensive if the material for printing is metal or alloy powder.

#### 2.1.5. Binder Jetting

Binder Jetting (BJ), is an additive manufacturing process developed at the Massachusetts Institute of Technology (MIT) during the 1990s [169], which was originally named Three-Dimensional Printing process (3DP) [170]. However, its commercialisation started in 2010 [171]. The materials handled by BJ are metals, alloys, ceramics, and even sand and glass [172]. Generally speaking, BJ employs two materials, the material from which the part is built (solid powder material) and the material that is used as a binder (liquid material) that helps the powder material to stick. In [173], a criterion is specified regarding what to pay attention to when selecting the binder:

- How the powders interact with the binder, taking into account wettability and penetration.
- The amount of binder residue in the cleaning process.

In addition, some BJ printers contain nozzles that can print in colour, from a color cartridge, allowing the production of multi-colour parts [4].

**Figure 11:**
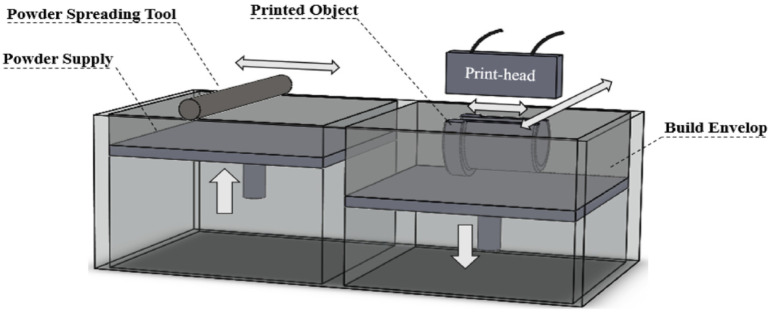
Illustration of the general BJ printing process. (Reprinted from *Additive Manufacturing*, Volume 28, Mohsen Ziaee and Nathan B. Crane, Binder jetting: A review of process, materials, and methods, 781–801, Copyright (2019), with permission from Elsevier).

The 3DP concept that belongs to BJ can be compared to PBF, in which a laser melts powder particles to gradually generate the layers of a part. The 3DP printing process consists in spreading the material to build the part with the use of a roller to achieve a thin layer of powder material so that a layer of the binder material can then be deposited where needed (as dictated by the STL obtained from the CAD model). This process is repeated till the part is complete [174]; see Figure 11. Nevertheless, in BJ printing processes, post-processing is performed after printing. This process can be related to curing, dust removal, or finishing, among others [172,175]. Post-processing is time-consuming, and a good example is that the powder structure is not strong enough to be used directly, so it needs to be infiltrated by a resin in order to densify the matrix [176]. This infiltration provides an improvement in colour definition and mechanical behaviour [177].

The principal advantages (+) and disadvantages (−) of BJ technology are:

+ The process has a very high speed compared to others.
+ It is able to process different kinds of materials.
+ 3D objects can be made in different colours.
+ With the two-material method, the printing can obtain different binder-powder combinations, as well as several mechanical properties.
− It is high in cost.
− It is not suitable for any structural parts because of the use of binder material.
− The post-processing could considerably increase the time of the whole process.

During printing, the height of the powder bed along the *z*-axis determines the layer thickness. In the case of BJ, the thickness can be between 15 and 300 μm. Therefore, the layer thickness is given by the desired resolution and ultimately by the powder size [178]. It should be noted that BJ has lower mechanical properties than SLS and DMLS parts [179]. However, BJ can manufacture complex lattice structures at much higher speeds [180].

As in PBF printers, the price of BJ printers is very high. The lowest price for a BJ printer can be around EUR 24,000 [181], and the highest price can be around EUR 500,000 [182]. Depending on the brand, the size of the construction area, and the type of material to work with, prices may also vary notably.

## 3. Important Features during the AM Process

AM has been a revolution in the field of fluid micromechanics, because it has managed to move away from the use of laborious techniques, such as soft lithography, which requires previous knowledge, several machines, expert manpower, many hours of work, and high investments of money for the correct manufacture of fluidic microdevices. Therefore, soft lithography lacks marketability due to the complexity of the whole process. The 3D printing technologies mentioned in the previous section, which include AM, propose an improvement in these disadvantages and offer machinery in different price ranges depending on the desired objectives along with the manufacturing of fluidic micromixers. Table 3, shows a collection of some of the fluidic microdevices that have been manufactured according to the literature.

### 3.1. Resolution and Precision

As aforementioned, the most popular AM categories are material extrusion, vat photopolymerization and material jetting. Depending on the chosen method, the results obtained are more or less useful for the desired purposes. The resolutions currently available in these categories are summarised in Table 4.

In order to give some recommendations according to resolution, we could say:

- High resolution values are quite good when the visual quality and the details are important. If the model has a complex geometry or is too small in material extrusion is necessary to use low layer heights to achieve more accuracy [200].
- Low resolution values are valid for rapid prototyping, in which the quality of the part is not the main factor. In some designs, there is no difference between 20 μm or 30 μm of layer thickness but there are in time and cost.
- If the model is going to be polished or sanded, or even painted (post-processing) after the printing, the resolution may be less relevant due to the fact that the layers will disappear.

In the following section, some references that could help to make decisions about the technology that fits best to achieve specific targets for each application will be given. It is important to note that some of the present-day printers that enable the user to have higher resolution did not exist a few years ago. Moreover, the resolution of the printer, which is linked to the resolution of the technique and vice versa, is not directly equivalent to the minimum wall thickness of the model to be printed. This is because there are some factors, mainly related to printing problems and post-processing, that could affect these dimensions, for instance, to choose the wrong orientation for printing or to damage the piece in the process of withdrawing from the printing bed [201].

Macdonald et al. [183] made a comparison of three types of technologies: FDM, Polyjet, and DLP. The aim was to check whether all of them were valid for the manufacture of a microdevices and to assess their performance as micromixers. The conclusion was that the used FDM printer was the cheapest and more suitable for manufacturing low-cost micromixers, because of the prices of the printer and the material, which are lower than those of the other technologies. However, the surfaces obtained with FDM were much rougher than with the other methods. This was expected because it is known that in the specifications of FDM, the precision at microscale is low. Polyjet technology offers the possibility of printing a larger number of devices at the same time and the generation of smaller channels (up to 200 μm), but the manufacturing times were larger, and the difficulty of removing the support material made the post-processing complicated. DLP was the technology that could provide the shortest post-processing times, and the quality of the model was similar to that of Polyjet. However, unfortunately, it was not able to produce microchannels as small as PolyJet did. In [184], a comparison was made between a device fabricated in Polyjet, SLA, and FDM. As in the previous work [183], the most accurate technology was Polyjet. FDM, due to its low precision compared to the others, made a smaller-channel fabrication compared to the designed CAD model. Moreover, the three methods were able to build valid micromixers, remarking that FDM and Polyjet behaved better with low flow rates, and SLA had the same behaviour for both low and high flow rates. In [194], the accuracy at different layer thicknesses was compared in models printed with an SLA and a DLP printer. It is interesting to highlight that by using a layer thickness of 100 μm, the DLP printer was faster and reached a higher accuracy compared to the SLA printer. The investigation carried out in [195] reports results of test structures printed with four different MJ 3D printers: Projet3000HD+, Projet3510SD, Objet24, and Objet30Pro. It was also determined that the minimum dimension that the printers could properly print for a microfluidic structure was 200 μm. Nevertheless, all the chosen printers had a nominal resolution of one order of magnitude better. Moreover, a comparison was done of the surface roughness of the best printouts, which was better than the surface roughness obtained when using a FDM printer. In addition, in their work, they proposed a method that aimed at correcting the mapping error. Aided by this method, it was possible to achieve a decrease in differences between CAD dimensions and real dimensions of less than 5%.

Obviously, precision and resolution are two specific values that are involved in printing performance. The investigation done by [135] determined the measurement range that the ProJet HD 3500 printer (Multijet) could print, ranging from 100 to 1000 μm in width and 50 to 500 μm in height. Values smaller than 50 μm and 100 μm in width and height, respectively, could not be included in the above ranges because the channels collapsed. In addition, the width between the CAD model and the 3D printed model varied around 35 μm in width and 11 μm in height. It has to be highlighted that it was observed that printing in the vertical direction gave better results in terms of accuracy. Lee et al. [185] used an Object Eden 350V (Polyjet) printer and a Dimension Elite (FDM) printer to evaluate microfluidic characteristics. They observed with the Polyjet printer an average deviation of 25.2 μm between the diameter of the CAD design and the 3D printing. Meanwhile, a higher average deviation of 67.8 μm was observed with the FDM printer. It was also concluded that the greater the sidewall angle, the greater the roughness. Additionally, the surface roughness had a value of 0.47 μm using Polyjet and 42.97 μm with FDM, which means smoother surfaces with the Polyjet technology. In [202], it is demonstrated that a variety of structures, consisting of embedded and open channels arranged in several different orientations with various sizes and printed in different types of resin, can be printed with the Form 2 printer. It is highlighted that there is a close relationship between accuracy, channel size, angle, and chosen resin. Furthermore, it is established that 500 μm is the printing limit for embedded channels and 250 μm for open channels. With the aim of finding quality ranges to obtain professional 3D printing, dimensional accuracy, flatness error, and surface texture were carried out in the study by Nuñez et al. [191], where different parts were manufactured with an FDM printer (Dimension Elite 3D Printer) using the ABSplus material. Thanks to these parts, it was possible to determine the tolerance and surface finishes that the machine could provide, obtaining better dimensional behaviour by using a layer thickness of 254 μm and an interior fill of 100%. In addition, the best surface finish and minimum flatness error were obtained using a layer thickness of 178 μm and 100% infill.

In [197], it is specified that the ability to fabricate thin walls is crucial for the performance of heat exchangers. Therefore, the fabrication of thin walls using the EOS M 290 printer is studied by PBF printing. The minimum wall thickness achieved was 100 μm in Ti-6Al-4V, Inconel 718 and AlSi10Mg. On the other hand, in [173] printing with BJ, researchers determined that a large particle size is beneficial for powder spreading. However, if the size is smaller, this is good for sinterability, and it has the advantage of improving the resolution and surface quality of the part.

### 3.2. Material

The used material is another important feature. In particular, the choice of the building material will be of a great importance if it is necessary to visualize the fluid mixing in a micromixer. Despite choosing a transparent material, the ability to work well as a transparent medium for fluid visualisation depends on the chosen technology and the desired surface finish. Device surfaces have to be smooth and free of defects, even with a transparent and colorless material. On the contrary, a 3D model printed with rough surfaces will appear translucent [186]. Notwithstanding, the appearance of the devices could be enhanced by treating devices during the post-processing stage [203], but when dealing with microdevices, this work can be cumbersome. One of the major limitations of all SLA printers is that they cannot use more than one printing material at a time. Thus, Choi et al. [204] developed a printer model with SLA technology that provided the multi-material printing using four different resin baths. Nonetheless, the process was complex and inefficient, as each layer of each resin required multiple exposures. In relation to the process of the removal of the support material, since this may influence the final result; this is another important feature to take it into account when selecting a printing method. It can be done in different ways:

- Material extrusion provides two options to remove supports:
  − Using a soluble support material [205]: an automated support-material process.
  − Breaking support material [85]: a manual process involving twisting, scrapping, and breaking support material from the printed part—it is useful to have pliers.
- Vat photopolymerization uses less support material than the others. Support material can be easily removed with water jets [120].
- Material jetting has different possibilities depending on the selected printer [195]:
  − Polyjet: use of a water jet to remove the support material.
  − Multijet: used to melt away support material at approximately 60 °C.
- Powder bed fusion uses support structures that, ideally, should be weak enough to be removed easily with minimal effort for cutting, breaking, or machining [206] but still provide stability to the part during printing.
- Binder jetting does not need support structures, since the remaining loose powder in the bed acts as support structure for the overhanging structures [207].

Moreover, the technologies associated with material extrusion do not always need to use support material. This depends on the design of the 3D model and on the model orientation that has been established with the slicer software. It is the same with the vat photopolymerization category. On the contrary, material jetting always uses support material. Therefore, Polyjet technology always has costs related to processing of support material removal. Consequently, for 3D printing of micromixers, Polyjet printers have limitations when manufacturing channels of a certain size, since the support material used to deposit the desired construction material must be removed. Material removal can be affected by the complex design of the micromixer model (right angles or even serpentine channels) [187]. If support material is not removed entirety, blockages in the channel can occur while the device is being tested [134]. Stratasys is a company that offers different types of support material, although not all of them are compatible with all Polyjet printers. However, amongst other options, it allows choosing a soluble support material, e.g., SUP706, when the printed model is introduced in a specific solution [137]. This avoids the risk of breaking certain delicate parts of the micromixer due to its size, instead of having the only option of using waterjets to remove the support material.

The aforementioned research has discussed printed microdevices in a single step to use them for different applications. Despite this, in [132], the authors tried to fabricate a microreactor with Polyjet, but instead of fabricating it in a single step, it was done in two steps, with one of the parts manufactured with a transparent material in order to ensure visible control. The printing process was completed in three hours, significantly shorter than the traditional approach with metal machining. The use of Polyjet made it possible to create channels up to 0.25 mm in diameter, as well as to create non-straight and zig-zag channels, extending the length of the channel, and increasing the fluid mixing. Furthermore, it is mentioned that using Polyjet, the roughness is usually approximately 1 μm, but in this case, due to the contact between the two printed parts, the contact surface was made glossy with the finishing parameters of the Polyjet machine, resulting in a reduction in roughness of 0.566 μm.

Additionally, a showerhead mixer was 3D printed in [189] with BJ in a matrix material of 316 stainless and bronze, and with DMLS in 316L stainless steel. Results show that the mixer enables high mixing performance.

### 3.3. Properties

Material properties are another important feature in AM of micromixers. Each technology offers different materials, some of which may be common in different technologies. On the one hand, the most well-known FDM technologies are ABS and PLA. ABS provides strength and some flexibility. PLA is biodegradable and shrinks less than ABS when cooled. However, PLA is less durable than ABS, is susceptible to heat, and, like ABS, is degraded by moisture in the air. Thus, for engineering parts, using ABS is more recommended. Another interesting material that can be used with this technology is ULTEM [208], which is a family of PEIs that are biocompatible and highly durable and have good thermomechanical properties (heat and wear resistant). Additionally, the authors of [192] assessed how the used angle and layer thickness could affect the results of the mechanical properties of the 3D piece printed with PEEK material. The optimal mechanical properties were found with a layer thickness of 300 μm and an angle of 0º/90º, which had a great impact on tensile, compressive, and three-point bending properties. Moreover, it was observed that the piece printed with the PEEK material had better mechanical properties than the ABS one. It is shown that PEEK could be a promising material for many industrial applications. In addition, Torres et al. [193] studied how the heat treatment could affect material properties and reliability in a FDM printing with PLA material. It was proven that with this treatment type, an increase in strength and a loss in ductility could be achieved. However, if low levels of heat treatment are used, the strength is, there is no loss of ductility, and reliability is preserved. Furthermore, tensile properties were investigated in [190] by observing what effects the chosen layer thickness could have in pieces printed with PLA. They finally stated that the layer thickness had an impact on tensile strength and modulus, since when the layer thickness increases, the aforementioned properties decrease. On the other hand, vat photopolymerization technologies offer smooth finishes with different types of resins [111]. Each resin has different mechanical and chemical properties: standard, structural, tough and durable, elastic, flexible, etc. Additionally, if it is necessary to improve the properties of these resins, additives can be incorporated into the photopolymer resin [209]. In addition, to obtain more advanced material properties, an interesting application of vat is multimaterial vat polymerization, which can provide materials with higher impact absorption and resistance to fracture [210]. An example is [211], where methacrylate-based commercial resins with carbon fibre additives are used, obtaining microlattices of high stiffness but also energy dissipative as elastomers.

In Polyjet, the properties that the material can achieve are less limited, as the number of combinations of printing materials is greater in order to create new materials with better properties. This results in materials with smooth surfaces and enhanced properties. A good example of this is shown in [188], where authors successfully printed 3D microfluidic valves. All valves were printed with the Objet500 Connex (Polyjet) printer, which had the capability of multimaterial printing. The valves were manufactured by combining a very flexible material and a rigid one: TangoPlus, a translucent material that is similar to rubber, and VeroWhitePlus, an opaque white material that is rigid. Several valve impressions were made using different ratios, achieving different values of tensile/tear strength and translucency for each device. The printed valves were demonstrated to work correctly.

In addition, in [198], with BJ technology, an optimization method was used to find the optimal parameters to improve the transverse rupture strength by determining the binder saturation (70%), layer thickness (100 μm), roller speed (6 mm/s), and feed-to-powder ratio (3). Moreover, in [212], also using BJ technology, alumina parts were fabricated, and parameters such as layer thickness, particle size, and sintering profile were alternated, resulting in parts with a relative density of 96%. Furthermore, it was determined that the use of increased powder distribution and lower layer thickness provided better results. In [213], the influence of layer thickness and part orientation on the mechanical properties was assessed and tested with tensile tests. The results showed that the thickness of the layers affects the mechanical properties during bonding to a greater extent than the orientation. However, after bonding and curing, the density directly affects the properties. On the other hand, in [214], eleven PBF printings were carried out, in which the properties of the recycled powder were evaluated. In the results, they found that the recycled powder did not show significant changes in its particle size. However, the density of the powder bed increased.

### 3.4. Capacity and Leakage

Regarding printing capacity, if printer investment is not a problem (more than EUR 50,000), all the described categories can offer the user a medium–large printing area. However, if this is not the case, as illustrated in [215], while spending less amount of money (between EUR 10,000 and EUR 50,000), it will be possible to achieve a larger printing capacity with material extrusion and material jetting printers than with vat photopolymerization printers. Meanwhile, if the investment has to be reduced, around EUR 3000, the only available options are material extrusion or vat photopolymerization, and taking into account the available dimensions of the printing area, material extrusion is the best, even though the dimensions will be similar between both if the printer cost is lower than EUR 3000. Furthermore, in relation to leakages, the technology that has greater risk of suffering this problem is generally material extrusion. This is so due to the way in which layers of the material extrusion printers are printed.

### 3.5. Cost

In reference to costs, in [216], the feasibility, versatility, and configurability of 3D printing was demonstrated during the fabrication of milli- and microreactors. In just one day, by means of a material extrusion printer, a 3D geometry was designed, printed, and used for synthesis reactions. The material used for printing was cheap and inert in order to avoid undesired reactions. This study demonstrated that this fabrication method is cost effective and affordable, which enables the design (inputs, outputs, and dimensions) to to be modified. Moreover, in [217], it was demonstrated that microfluidic devices that enable the incorporation of electrodes can be fabricated with a low-cost 3D printer. Printing was carried out by a Form1+ printer, which is a type of stereolithography technology, and a transparent resin was used for printing, which facilitated the visualisation of the electrodes located inside the channels. This investigation can be considered as a basis for the fabrication of other more sophisticated, but low-cost, devices. A good example of comparison between technologies in the literature is [205]. In this work, a comparison of printing accuracy between a FDM and a Polyjet printer is made. They observed that Polyjet is the one that exhibited higher accuracy to reproduce the desired replica. Although the differences are not so great with respect to the replica obtained with the FDM printer, it should be noted that the investment made in the FDM printer is greater than in Polyjet. For example, the price of Fortus 250mc is higher [218] than that of Objet30 Pro [219]. Nevertheless, in the case in which the price range has to be lowered, e.g., between EUR 600 and EUR 2500, Valentin et al. [220] presented a printing process that enables the creation of transparent devices with FDM. The aim was to correctly visualise fluid interactions, along with an attempt to achieve an efficient microfluidic device using low-cost printers. Furthermore, it was noted that despite achieving maximum resolution, corners forming a 90º angle were impossible to recreate with the chosen configuration, due to the circular shape of the nozzle, which led to rounded profiles. Regardless of that issue, an efficient mixing and water-free leakage was obtained. The resolution limit assessed using different FDM printers in [220,221] agreed on the specified value, about 400 μm.

### 3.6. Assessment of Specifications

In order for the user to have an idea of how different specifications (which the authors think are of great interest) can be reached with each category, qualitative information is given in Table 5. It must be noted that the specifications shown in Table 5 are general, and some of them are adjusted to the maximum possibilities that the printers in each category can offer. Additionally, Table 6 shows some references classified according to the information provided on certain specific topics.

### 3.7. Slicers

Apart from the decision of the printing technology and the printer, it is necessary to select the software of the 3D slicer, because within the 3D printing workflow, it is essential to use not only a CAD program but also a 3D slicer software. This is the intermediary between the 3D model and the printer; see Table 7. A wide variety of CAD programs are now available to create multiple designs and to specify their corresponding dimensions. The same happens with slicer software; there are a large range of options that offer many possibilities to set down the printing parameters with which it is desired to print the design made. Slicers are not strictly classified as Computer Aided Manufacturing (CAM) systems [224]. However, they work to achieve the same objective in the process of 3D printing: they convert its inputs in digital files that contain detailed instructions for printers. Thus, when the design is completely finished in the CAD program, the file is saved in Standard Tessellation Language or Stereolithography (STL) format and is used as input [225] for the chosen slicer software. Moreover, the STL file is a triangle mesh that represents the 3D object that has been designed. Data of this triangular mesh are suitable for providing a 3D model description [226]. Next, when different print parameters have been specified to the slicer, as a final step, the piece is sliced and is saved in G-code format. The G-code will give the instructions to the printer: indications on where to move, how fast, and even what trajectory to follow [227]. There is a large variety of slicers in the market, and many are available for free [228]. One of the most popular is Cura, which is free, and it enables users to develop external plugins. It is suggested to beginners and semi-professionals, but there is a professional version for companies, which has an annual cost. Furthermore, PrusaSlicer is a valid software for FDM, SLA, and DLP technologies, and it is also free. ChiTuBox and PreForm are also free. Nevertheless, common slicer softwares of popular material jetting printers are not free, since these kinds of printers are usually found for industrial processes. Normally, when using an industrial printer, 3D models have to be sliced by a specific proprietary software [229,230].

There are many slicers with different advantages; however, they also have disadvantages. This tool has a major impact on the final result of the printed model. Therefore, a good slicer should not only calculate each layer individually but also take into account the top and bottom layers. Furthermore, having good slicers is of great importance for complicated structures and areas with overhangs [231,232].

The authors of [196] assessed and compared the cost and print time estimation for six 3D printers with their corresponding slicer softwares. When using Cura LulzBot Edition 3.6.20 for LulzBot TAZ 6 (FDM), PrusaSlicer 2.2.0 for Prusa i3 MK3S (FDM), GrabCAD Print—Version 1.43 for Stratasys F370 (FDM), PreForm 3.4.6 for Formlabs Form 2 (SLA), and PrusaSlicer 2.2.0 for Prusa i3 MK3S (SLA), the models to be printed are automatically placed in the centre of the printing bed. However, for GrabCAD Print—Version 1.43, used with the Stratasys J750 Digital Anatomy (Polyjet), and PreForm 3.4.6, used with the Formlabs Form 3 (SLA), the models to be printed are automatically placed in the corner of the printing bed. The location of the models does not influence the printing time and the amount of used material, but in the case of the Stratasys J750 Digital Anatomy it does, because GrabCAD Print—Version 1.43 places the models so as to minimize printing time and material used. Moreover, it was observed that a horizontal orientation of the model minimizes the printing time, whereas a vertical orientation minimizes the cost, although horizontal orientation o the Stratasys J750 Digital Anatomy minimizes the cost. In addition, if low values of layer height are used, the printing time increases, but the cost decreases (with the exception of the Stratasys F370). Baumann et al. [231] and Šljivic et al. [233] tested how different slicers for FDM technology could influence the final result of the printed model. All the tests were developed using the same setting, and slicers were evaluated according to the smoothness of the surfaces, the achievement of fine structures, the quality of the overhangs, the precision, the speed, and the usability. On top of that, it is specified in [231] that the results obtained could be different if a different printer is used than the one used in this research.

In [221], a comparison was made between different types of slicers to determine the fidelity and performance in relation to the printing of a micromixer. During their investigation, researchers used an FDM low-cost printer, and its limitations were also evaluated. Additionally, Tamburrino et al. [83] specified the effect of slicing parameters on the printing performance with an Ultimaker 3 printer and the Cura 3.1.0 software as the slicer.

## 4. Future Prospects in AM of Micromixers

As AM is poised to become the most widely used technology for the manufacturing of micromixers and other microfluidic devices, as well as for many other applications in other fields, it must continue enhancing the possibilities currently offered by the different technologies available on the market.

Each 3D printing technology has some room for improvement. For instance, FDM technique, which is widely used for multiscale and multimaterial printing of polymer composites, has a limited structural fidelity and mechanical properties of the printed components. PolyJet has high costs in terms of equipment, as do PBF and BJ categories. SLA, DLP, and even CLIP, although capable of rapidly producing structures with good resolution, they can print only with a limited number of materials and for small printing volumes. Moreover, fabrication of multimaterial structures using vat photopolymerization techniques remains a challenge [234]. Other 3D printing specifications will also need to be improved, such as achieving smaller levels of resolution, as well as higher levels of precision and accuracy, to enable the manufacturing of closed microchannels down to 10 μm, which can be produced in a wide range of materials [93]. Furthermore, researchers are committed to continuing to advance the development of other polymeric materials for AM technologies. This allows them to achieve better functionalities in both electrical and thermal conductivity, and in biocompatibility. Moreover, it is of strong interest to further develop multifunctionality [235] and localised functionality capabilities with the help of shape-changing and smart materials [209] such as piezoelectric, electro-rheological fluid, and shape memory alloy, among others [236]. In addition to the above, the use of AM technologies is still too costly in many cases [237], and the post-processing process sometimes becomes tedious.

It is interesting to think about the extended use of smart materials for printing purposes. Smart materials are materials that respond to external stimuli such as heat, moisture, stress, pH, or magnetic fields, and they can be 3D printed. This type of printing is called 4D printing [238]. 4D printing would be very useful in advanced micromixing applications developed in computational studies to date. Clear examples of advanced applications only feasible in AM by means of 4D printing are shown as follows. Shoele and Mittal [239] placed a flexible reed inside a heated channel, whose movement followed a fluttering motion due to the flow that goes throughout the channel. This was used to study the relationship between the aeroelastic vibration of the reed and heat-transfer enhancement. Gallegos and Sharma [240] enhanced heat transfer using “flags” for the generation of vortices within a channel, demonstrating that it was a successful technique. However, it is remarked in [240] that the used mechanism has not been tested very much and that there is a need for experimental studies to validate the theoretical results already obtained, which could be only addressed in AM by means of 4D printing. In [241], instead of using one flag, two flexible flags were used in order to analyse and study the dynamics of the flags and the effects they had on the enhancement of heat transfer, even on the resulting vortices. The flag flutter dynamics and heat transfer properties were also analysed. These flags were also located inside a heated channel as well as in the research mentioned above. Additionally, the authors of [242] assessed a channel that has a cylinder with a flexible plate. Hence, the vortex shedding created by the cylinder in the channel results in the plate oscillating downstream. In this way, Vortex Induced Vibration (VIV) is introduced with the aim of altering the thermal boundary layer to increase heat transfer rate.

In conclusion, it is of growing interest in the AM field to continue evolving to fill those gaps in micromixing applications where there is a lack of experimental studies due to manufacturing restrictions and further expand the applicability of this manufacturing technology. This of course can be extendable to other aspects such as quality, post-processing, and cost, as discussed above, which also need further improvement.

## 5. Conclusions

AM of microdevices such as micromixers has gained a lot of interest in recent years. However, this manufacturing field is still unknown for many researchers, as it is a relatively new technology. The present review has been focused on the discussion of the pros and cons of existing 3D printing technologies, as well as a deep discussion of their individual characteristics in terms of price, resolution, precision, and materials for the development of micromixing machines.

In this work, it has been concluded that the material extrusion category is the cheapest, as opposed to material jetting, powder bed fusion, and binder jetting categories, which are the most expensive. The quality of the printed micromixer in terms of precision and resolution are the best, with vat photopolymerization and material jetting categories. Regarding the materials used to construct micromachines, the best one is material jetting because of the fact that material jetting printers have the capacity to print with different materials at the same time and to combine them to obtain specific colours, textures, and properties. Moreover, all the categories offer printers with huge capacity, although this is not the case when considering leakages, in which the worst category is the material extrusion technology. In addition, when working with fluids at microscale to manufacture micromixers, it is important to highlight other aspects such as resolution, material (transparent materials for visualisation), leakage, and the support material removal procedure (especially for embedded microchannels). Hence, by considering all these aspects, the technology that may be the best is Polyjet if more importance is attributed to resolution and material. However, if price is of greater concern, then FDM technology is the best, whereas if speed and resolution are relevant, SLA or DLP technologies are better options for printing the desired micromixer. Additionally, in the event that the desired material is metal, the best option would be to use 3DP technology, since it is not necessary to remove the support structure as it does not need any. Hence, there is no concern regarding post-processing times. Notwithstanding, the size of the micromixer cannot be very small due to the minimum layer thickness that this technology can print. In addition, the technology chosen cannot be the SLS, because of the fact that it does not work well with wet parts.

From all the information reviewed in this work, it can be stated that there is no single best technology for fabrication of fluidic micromixers. The best technology to be used will depend on the design of the device, the desired performance, and requirements of each case. However, as AM is becoming more and more widely used, the authors of this review foresee an increasing importance and applicability of AM in the construction of microdevices with advanced performance. Thus, there will be continuous major advances in order to meet certain needs that will continue to improve manufacturing of micromixers: higher resolutions, smart materials with enhanced properties, easier post-processing methods, etc.

## Figures and Tables

**Table 1:** Classification of 3D printing technologies according to the ASTM [17].

Categories	Technologies	Materials
Binder jetting	3D printing	Metal, Polymer, Ceramic, Sand
Direct energy deposition	Laser Engineered Net Shaping, Electron Beam Additive Manufacture	Metal powder: steel, titanium, …
Material extrusion	Fused Deposition Modeling, Direct Ink Writing	Polymer, Hydrogel, Alloy, Pure metal
Material jetting	Polyjet, Multijet, NanoParticle Jetting	Photopolymer, Wax, Metal, Ceramic
Powder bed fusion	Selective Laser Sintering, Selective Laser Melting, Direct Metal Laser Sintering, Electron Beam Melting, Selective Heat Sintering	Metal, Polymer, Ceramic
Sheet lamination	Ultrasonic Consolidation, Laminated Object Manufactured	Hybrids, Metal, Ceramic
Vat photopolymerization	Stereolithography, Digital Light Processing, Liquid Crystal Display, Continuous Liquid Interface Production, Two-Photon Polymerization	Photopolymer, Ceramic

**Table 2:** Temperatures for the processing of the common FDM materials.

Materials	Bed Temperature [°C]	Extrusion Temperature [°C]
PLA	60–90	175–220
ABS	80–100	230–260
PET-G	50–80	220–260
ASA	90–110	250–280
PP	60–110	220–260

**Table 3:** Some examples of the application of AM technology in microfluidics.

References	Technology	Fluidic Structure
[132]	Polyjet	Microreactor: Y-shaped
[134]	Polyjet	Enclosed microfluidic channels: serpentine and Y-mixers
[135]	Multijet	Microfluidic circuitry
[137]	Polyjet	Multimaterial microfluidic capacitor
[183]	FDM, DLP and Polyjet	Y-junction microfluidic device
[184]	FDM, SLA and Polyjet	Classical Y-shaped connected channels
[185]	Inkjet and FDM	Microfluidic chip
[186]	DLP	Water-impermeable, biocompatible, transparent and cheap devices
[187]	Polyjet	Phantom of a right internal carotid
[188]	Polyjet	Multimaterial microfluidic proportional valve
[189]	DMLS and Binder jetting	T-mixers into a laminated “showerhead” structure
[165]	SLM	Milli-reactor

**Table 4:** Resolutions/layer thickness available depending on the 3D printing category.

	Material Extrusion	Vat Photopolymerization	Material Jetting	Powder Bed Fusion	Binder Jetting
	[190,191,192,193]	[183,184,194]	[97,183,195,196]	[165,197]	[173,198,199]
	500				
	300	100	30	700	200
Resolution/layer thickness [µm]	200	50	16	400	100
	100	25	14	200	80
	50				

**Table 5:** 3D printing category specifications that best suit (⋆⋆⋆), (⋆⋆) intermediate suit or not (⋆) the uses.

Specifications	Categories				
	Material Extrusion	Vat Photopolymerization	Material Jetting	Powder Bed Fusion	Binder Jetting
Printing time [222]	⋆⋆	⋆	⋆⋆⋆	⋆	⋆⋆
Precision	⋆	⋆⋆	⋆⋆⋆	⋆⋆⋆	⋆⋆⋆
Resolution	⋆	⋆⋆	⋆⋆⋆	⋆	⋆
Materials (variety)	⋆⋆	⋆	⋆⋆⋆	⋆⋆	⋆⋆⋆
Material (transparency issues)	⋆	⋆⋆	⋆⋆⋆	⋆	⋆
Capacity of the printing bed	⋆⋆⋆	⋆⋆⋆	⋆⋆⋆	⋆⋆	⋆⋆
Leakage [165]	⋆	⋆⋆⋆	⋆⋆⋆	⋆⋆⋆	⋆⋆⋆
Printer price	⋆⋆⋆	⋆⋆	⋆	⋆	⋆
Material price	⋆⋆⋆	⋆⋆	⋆	⋆	⋆

**Table 6:** Classification of references used in the text according to certain topics covered.

Specifications	Categories				
	Material Extrusion	Vat phOtopolymerization	Material Jetting	Powder Bed Fusion	Binder Jetting
Resolution, precision and accuracy	[190,191,192,193] [183,184,200,220,221]	[183,184] [194,202]	[97,135,183] [184,185,195]	[165] [197]	[173,178] [198,199]
Materials	[85,190,192] [187,193,205,208]	[111,116,120] [186,204,223]	[132] [137,188]	[149,150,165] [189,206]	[4,172,173] [189,207]
Prices	[88,89,216,220]	[122] [123,217]	[96,145,146]	[167,168]	[181,182]

**Table 7:** Printer brands with popular slicer softwares.

	Categories				
	Material Extrusion	Vat Photopolymerization	Material Jetting	Powder Bed Fusion	Binder Jetting
Popular printer brands	Ultimaker, bq, MakerBot, BCN3D, Creality, Stratasys, etc.	3D Systems, Nexa3D Formlabs, Miicraft, etc.	Stratasys, 3D Systems	3D Systems, EOS	ExOne, VoxelJet
Popular slicers	Cura, PrusaSlicer	PrusaSlicer, ChiTuBox, PreForm	GrabCAD Print, 3D Sprint, 3DXpert	3DXpert, EOSPRINT 2	3DPrinterOS

## Abbreviations

3D	Three-dimensional
AM	Additive Manufacturing
CAD	Computer-Aided Design
RP	Rapid Prototyping
ASTM	American Society for Testing and Materials
PDMS	Polydimethylsiloxane
ABS	Acrylonitrile Butadiene Styrene
PA	Polyamide
PC	Polycarbonate
PEKK	Polyetherketoneketone
PLA	Polylactic Acid
PPSF	Polyphenylsulfone
TPU	Thermoplastic Polyurethane
SLA/SL	Stereolithography
UV	Ultraviolet
DLP	Digital Light Processing
LCD	Liquid Crystal Display
CLIP	Continuous Light Interface Production
2PP	Two-Photon Polymerization
PE	Polyethylene
PP	Polypropylene
EPU	Elastomeric Polyurethane
MJ	Material Jetting
DOD	Drop On Demand
CIJ	Continuous Ink Jet
NPJ	NanoParticle Jetting
CAM	Computer Aided Manufacturing
STL	Standard Tessellation Language
CCD	Charge-Coupled Device
PEI	Polyetherimide
LOC	Lab-On-a-Chip
VIV	Vortex Induced Vibration
PET-G	Polyethylene Terephthalate with Glycol
PP	Polypropylene
ASA	Acrylonitrile Styrene Acrylate
CFD	Computational Fluid Dynamics
PBF	Powder Bed Fusion
SLS	Selective Laser Sintering
SLM	Selective Laser Melting
DMLS	Direct Metal Laser Sintering
SHS	Selective Heat Sintering
EBM	Electron Beam Melting
BJ	Binder Jetting
3DP	Three Dimension Printing
